# Interaction between Fibronectin and β1 Integrin Is Essential for Tooth Development

**DOI:** 10.1371/journal.pone.0121667

**Published:** 2015-04-01

**Authors:** Kan Saito, Emiko Fukumoto, Aya Yamada, Kenji Yuasa, Keigo Yoshizaki, Tsutomu Iwamoto, Masahiro Saito, Takashi Nakamura, Satoshi Fukumoto

**Affiliations:** 1 Division of Pediatric Dentistry, Department of Oral Health and Development Sciences, Tohoku University Graduate School of Dentistry, Sendai, Japan; 2 Operative Dentistry, Department of Restorative Dentistry, Tohoku University Graduate School of Dentistry, Sendai, Japan; 3 Pediatric Dentistry, St. Mary’s Hospital, Kurume, Japan; 4 Section of Orthodontics, Division of Oral Health, Growth and Development, Faculty of Dental Science, Kyushu University, Fukuoka, Japan; 5 Department of Pediatric Dentistry, University of Tokushima Graduate School, Tokushima, Japan; Seoul National University, KOREA, REPUBLIC OF

## Abstract

The dental epithelium and extracellular matrix interact to ensure that cell growth and differentiation lead to the formation of teeth of appropriate size and quality. To determine the role of fibronectin in differentiation of the dental epithelium and tooth formation, we analyzed its expression in developing incisors. Fibronectin mRNA was expressed during the presecretory stage in developing dental epithelium, decreased in the secretory and early maturation stages, and then reappeared during the late maturation stage. The binding of dental epithelial cells derived from postnatal day-1 molars to a fibronectin-coated dish was inhibited by the RGD but not RAD peptide, and by a β1 integrin-neutralizing antibody, suggesting that fibronectin-β1 integrin interactions contribute to dental epithelial-cell binding. Because fibronectin and β1 integrin are highly expressed in the dental mesenchyme, it is difficult to determine precisely how their interactions influence dental epithelial differentiation *in vivo*. Therefore, we analyzed β1 integrin conditional knockout mice (*Intβ1^lox-/lox-^/K14-Cre*) and found that they exhibited partial enamel hypoplasia, and delayed eruption of molars and differentiation of ameloblasts, but not of odontoblasts. Furthermore, a cyst-like structure was observed during late ameloblast maturation. Dental epithelial cells from knockout mice did not bind to fibronectin, and induction of ameloblastin expression in these cells by neurotrophic factor-4 was inhibited by treatment with RGD peptide or a fibronectin siRNA, suggesting that the epithelial interaction between fibronectin and β1 integrin is important for ameloblast differentiation and enamel formation.

## Introduction

Tooth development is regulated by epithelial–mesenchymal interactions, leading to differentiation of the dental epithelium into enamel-forming ameloblasts and of the dental mesenchyme into dentin-forming odontoblasts. During this interaction process, growth factors and extracellular matrices are important for the determination of tooth size and shape. The basement membrane component laminin α5 was reported to regulate proliferation of the dental epithelium, and disruption of laminin α5 was found to result in small tooth size and lack of cusp formation [1].

Changes in the extracellular matrix (ECM) accompany developmental events such as cell migration, differentiation, and morphogenesis [2–4]. Fibronectin is an adhesive glycoprotein of the ECM that functions in cell adhesion, differentiation, and growth. Fibronectin associated with the dental basement membrane appears to play a crucial role in the terminal differentiation of neural crest-derived dental mesenchymal cells and regulates the elongation and polarization of odontoblasts through interactions between its transmembrane domain and the cytoskeleton [5–7]. Furthermore, fibronectin is present in the odontoblast layer during both the embryonic and adult stages of human dentinogenesis [8], suggesting its possible roles in the migration of odontoblasts to the pulp and in the maintenance of dentinogenesis. In addition, fibronectin is spatially and temporally distributed in conjunction with the enamel protein amelogenin [9]. However, expression of fibronectin in the dental epithelium and its functions related to the adhesion, proliferation, and differentiation of the dental epithelium are not clearly understood.

Integrin heterodimers containing the β1 subunit are expressed by many cell types, and the subunit is associated with various other molecules [10]. The β1 subunit imparts ligand specificity, enabling the heterodimer to bind to specific ECM or basement-membrane components. The major adhesion receptor for fibronectin is α5β1 integrin. Studies of the fibronectin receptor α5β1 using cultured fibroblasts suggest that upon ligand engagement, the short cytoplasmic domain of β1 integrin binds to proteins, which in turn associate with and reorganize actin filaments that form focal adhesions. Upon further activation of Rho GTPases, changes in the actin cytoskeleton lead to integrin clustering, which facilitates the polymerization and assembly of ECM on the cell surface and enables stable substratum attachment [11, 12]. Deletion of β1 integrin in mice resulted in inner cell mass failure and peri-implantation lethality [13]. Conditional deletion of β1 integrin from cells of the skin and mammary epithelium established the dependence on β1 integrin-mediated cell adhesion of proliferation in these tissues *in vivo* [14–16]. However, the roles of integrins in tooth development remain poorly understood. We previously showed that α6, α3, and β4 integrins are highly expressed in the dental epithelium but not in the dental mesenchyme [1]. The α6β4 integrin molecule associates with the laminin α5 chain and regulates cell polarity in the dental epithelium via the Rac1 and Cdc42 pathways. In contrast to the β4 subunit, the expression of β1 integrin was shown to be weaker in the inner dental epithelium as compared with the surrounding dental mesenchyme and basement-membrane area. Abundant expression of β1 integrin has been detected in regions of the basement membrane and in mesenchymal cells throughout development, which is consistent with its association with multiple subunits [1, 17]. However, the role of β1 integrin in the differentiation of the dental epithelium is unknown, mainly because its expression is relatively low in the dental epithelium compared to the dental mesenchyme.

In the present study, we investigated the role of the β1 integrin–fibronectin interaction in tooth development. Fibronectin was found to be expressed in the inner dental epithelium, but not during the secretory (S) stage of ameloblasts, and then again during the late stage of maturation (LM). Conditional knockdown (CKO) of β1 integrin expression under the control of the cytokeratin-14 (*K14*) promoter caused enamel hypoplasia and detachment of ameloblasts during LM and the formation of a microcyst. Furthermore, delayed eruption of teeth and inhibition of cell adhesion to fibronectin were observed in mutant molars, while enhanced ERK phosphorylation and ameloblastin expression induced by the neurotrophic factor NT-4 was observed in dental epithelia cultured with fibronectin, indicating that the interaction between β1 integrin and epithelial fibronectin is essential for ameloblast differentiation.

## Materials and Methods

### Mouse Strain

B6;129-*Itgb1* ^*tm1Efu*^/J mice (Jackson Laboratory), hereafter termed *Itgβ1* ^*lox-/lox-*^, harbor *loxP* sites introduced into the flanking region of exon 3 of the β1 integrin gene. *Itgβ1* ^lox-/lox-^ mice were bred with cytokeratin 14 Cre (K14-Cre) mice to produce animals that were hemizygous for the *K14-Cre* allele, and were either homozygous (*Itgβ1*CKO) or heterozygous for the floxed *Itgβ1* allele. For detection of the allele containing *loxP* sequences flanking exon 3 of β1 integrin, Southern blot analysis was performed using the BamHI fragment of mouse genomic DNA and visualized by the ^32^P-labeled exon 3 fragment, as previously described [15]. The ages of the mice after birth (in days) are indicated by E (embryonic day number) or P (postnatal day number), e.g., E15 or P1. All animal experiments were approved by the Animal Ethics Committee of Kyushu University. Seventy-three mice and 6 pregnant mice were sacrificed by cervical dislocation under isoflurane anesthesia for real-time PCR, immunohistochemistry, micro-computed tomography (CT), and primary cell culture.

### RNA Isolation and Real-time PCR

Total RNA was prepared using TRIzol (Invitrogen) [18] from rat enamel organs at the S, early maturation (EM), and LM stages of maturation. First-strand cDNA was synthesized at 50°C for 50 minutes using oligo(dT) or random primers with the SuperScript III First-strand Synthesis System (Invitrogen). PCR was performed with SYBR Select Mastermix (Applied Biosystems) and the StepOnePlus Real-time PCR system (Applied Biosystems). Primers for fibronectin, amelogenin, and ameloblastin were prepared as previously described [19–21]. Primers for β1 integrin (NM_017022.2: forward 5′-TTGGTCAGCAGCGCATATCT-3′, reverse 5′- ATTCCTCCAGCCAATCAGCG-3′), β4 integrin (NM_013180.1: forward 5′-ATACCAGCTACTCAACGGCG-3′, reverse 5′-CCGTACCCGGAACACATAGG-3′), β5 integrin (NM_147139.2: forward 5′-CAGTGGAAGTGCCACCTCAT-3′, reverse 5′-CGAGAGATGATGGACCGTGG-3′), and β6 integrin (NM_001004263.1: forward 5′-GCTCAAGTTACTTTTCAAAGCAGT-3′, reverse 5′-GCCACCTTGGACGTGATCATT-3′) were used for real-time PCR. Expression of each gene was normalized to GAPDH (NM_017008.4: forward 5′-AAGGCTGTGGGCAAGGTCAT-3′, reverse 5′-CTGCTTCACCACCTTCTTGAC-3′) expression. The highest expression level observed in each experiment was set as 1.0, which was used to calculate relative expression levels of all other samples. Statistical analysis of gene expression was performed using the Student’s *t*-test.

### Cell Culture and Transfection

SF2 (rat dental epithelial cells) and mDP (mouse dental papilla cells) cell lines were established as previously described [22]. A primary culture of dental epithelial cells from β1 integrin CKO mice was separated from dissected P1 molars as previously described [20], which was followed by culture in DMEM/F12 medium at 37°C in a humidified incubator in an atmosphere containing 5% CO_2_. For inhibition of β1 integrin and fibronectin expression, short interfering RNAs (siRNAs) were transfected into cells using siRNAs for β1 integrin (Stealth siRNAs RSS302262, RSS302263, RSS302264, and Stealth RNAi siRNA Negative Control, Med GC) and fibronectin [19]. For blocking integrin-dependent cell adhesion, antibodies for each integrin were used as previously described [1].

### Preparation of Tissue Sections

To prepare head specimens, 6-week-old mice were anesthetized and their tissues were fixed by perfusion with 4% paraformaldehyde dissolved in phosphate-buffered saline (PBS). Mouse heads were dissected and fixed overnight at 4°C with 4% paraformaldehyde in PBS. Tissues were dehydrated using xylene and a graded ethanol series, and embedded in Paraplast paraffin (Oxford Laboratories). Paraffin-embedded sections (10 μm thick) were prepared using a microtome (Leica). For morphological analyses of molars and incisors, sections were stained with Harris hematoxylin and eosin Y (H-E) (Sigma-Aldrich).

### *In Situ* Hybridization and Immunohistochemistry

Digoxigenin-11-dUTP single-stranded RNA probes for detecting fibronectin mRNA were prepared using a digoxigenin RNA labeling kit (Roche). The sections were treated with proteinase K and acetic anhydride and overlaid with 150 μl of hybridization solution containing the digoxigenin-labeled fibronectin probe (1 μg/ml). Then, they were denatured at 70°C for 60 minutes and hybridized overnight at 65°C. Hybrids were detected with an anti-digoxigenin antibody conjugated to alkaline phosphatase (Roche). Sections were incubated in 1% bovine serum albumin/PBS for 1 hour before incubation with the primary antibody. Primary antibodies specific for fibronectin (kindly provided by K. Yamada), collagen IV [1], dentin sialoprotein (DSP) [20], ameloblastin [20], laminin β1γ1 (MAB1905; Merck Millipore), and β1 integrin (9EG7: BD Biosciences) were detected using Alexa488- or Alexa594-conjugated secondary antibodies (Invitrogen). Nuclei were stained with Hoechst dye (Sigma-Aldrich) or DAPI (Vector Laboratories). A fluorescence microscope (BZ-8000, KEYENCE) was used for imaging analysis. Images were prepared using a BZ analyzer (KEYENCE) and Adobe Photoshop (Adobe Systems, Inc.).

### Micro-CT and Analysis of Incisor Yellow Spots

For radiographic and micro-CT analyses, half mandibles were dissected from 3-week-old wild type (WT) and homozygous mice. Micro-CT analysis was performed by Kureha Special Laboratory (Iwaki, Japan). Images of incisors from 8-week-old mice were acquired using a stereomicroscope. The yellow areas of images were analyzed using Adobe Photoshop (Adobe Systems, Inc.).

### Cell Adhesion Assays

Cell adhesion assays were performed using 96-well round-bottomed microtiter plates (Immulon-2HB; Dynex Technologies, Inc.). The wells were coated overnight at 4°C with 10 μg/ml human fibronectin (GIBCO BRL), collagen I (Nitta Gelatin Co., Japan), laminin 111 (laminin 1; Invitrogen), laminin 211 (laminin 2; Invitrogen), and human laminin 511/521 (laminin10/11; R&D Systems), and then diluted with PBS and blocked with 3% bovine serum albumin for 1 hour at 37°C. After washing, 10,000 cells were added and incubated for 60 minutes at 37°C. Antibodies against β1, α6, and α4 integrins, or the RGD and RAD peptides were added to the culture medium as potential inhibitors of cell adhesion. The plates were washed 3 times with PBS to remove unattached cells, and then the attached cells were identified by staining with crystal violet and evaluated using spectrophotometric analysis (wavelength; 600 nm).

### Western Blotting

Cells were washed twice with 1 mM ice-cold sodium orthovanadate (Sigma-Aldrich) in PBS, lysed with Nonidet P-40 buffer supplemented with a proteinase inhibitor mixture (Sigma-Aldrich), and centrifuged, and then the supernatants were transferred to fresh tubes. Lysates were separated using 4–12% sodium dodecyl sulfate-polyacrylamide gel electrophoresis (NuPAGE: Invitrogen) and analyzed by western blotting. The blotted membranes were incubated with antibodies for β1 integrin (9EG7: BD Biosciences), ERK1/2, and phosho-ERK1/2 (Cell Signaling), and signals were detected with an ECL kit (Amersham Biosciences) and visualized using the ImageQuant LAS 4000 Mini image analysis system (GE Healthcare, Life Science).

## Results

### Fibronectin Expression during the Presecretory and Late Stages of Ameloblast Maturation

**Fig 1:**
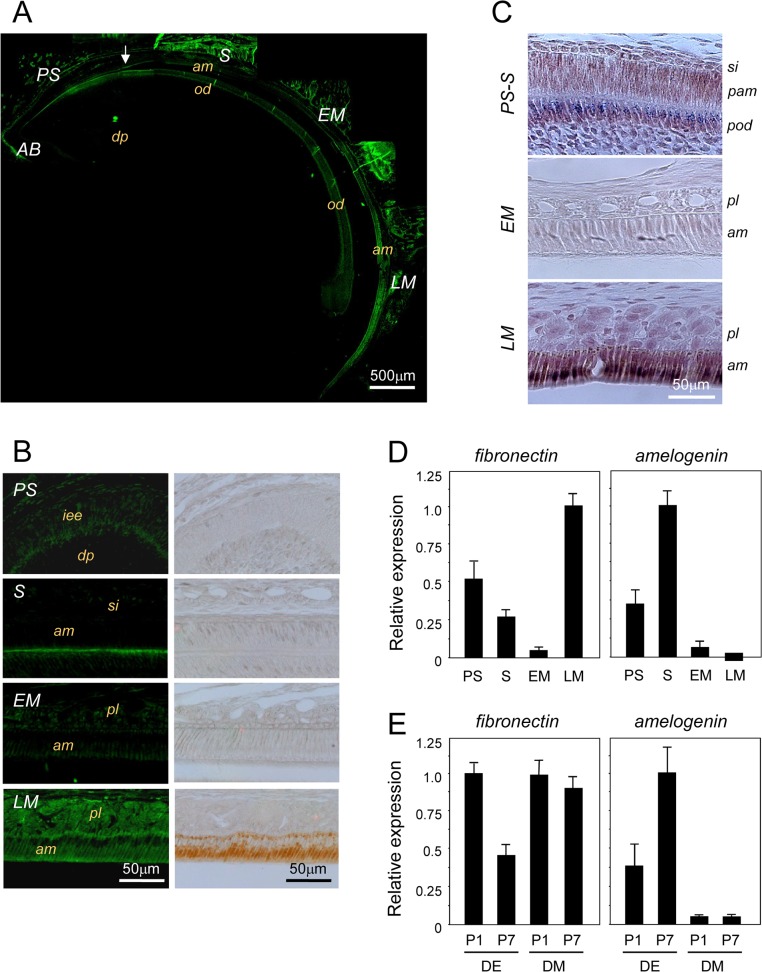
Analysis of fibronectin expression during tooth development. A. Immunofluorescence analysis of fibronectin expression using an anti-fibronectin antibody in sagittal sections from 6-week-old mouse incisors. The incisors were separated into the apical bud (AB), presecretory (PS), secretory (S), early maturation (EM), and late maturation (LM) stages. B. Immunofluorescence and immunohistochemical results are shown at a higher magnification. Fibronectin was expressed in the basal lamina at the S stage and in the papillary layer at the LM stage. C. Fibronectin mRNA was detected using *in situ* hybridization in the basal lamina, pre-odontoblasts, pre-ameloblasts from PS to S (PS-S) and LM ameloblasts, with lower expression seen during the EM stage. D. The expression levels of fibronectin and amelogenin were investigated by real-time PCR using PS, S, EM, and LM dental epithelial cells separated from the incisor. E. The expression of fibronectin in dental epithelial (DE) and dental mesenchymal (DM) cells isolated from the molars of P1 and P7 mice (n = 5) was investigated by real-time PCR. Amelogenin mRNA expression increased in DE cells, while fibronectin mRNA expression decreased with differentiation. iee, inner enamel epithelium; dp, dental pulp; si, stratum intermedia; am, ameloblasts; pam, pre-ameloblasts; od, odontoblasts; pod, pre-odontoblasts; pl, papillary layer.

To isolate the molecules important for tooth development, especially ameloblast differentiation, we previously performed differential display analysis of the genes expressed during 3 different stages of ameloblast maturation using mRNAs isolated from cells at each stage. Cadherin-related neuronal receptor family molecules were found to show stage-specific tooth germ expression [23]. Further, our results showed that fibronectin mRNA was specifically expressed during the presecretory (PS), S, and LM stages, but not the EM stage of ameloblasts (Fig. 1). Immunohistochemical analysis also showed that fibronectin was expressed in the PS stage of the dental epithelium. In the S stage, fibronectin was localized in the basement membrane. In the LM stage, fibronectin was detected both in ameloblasts and in the papillary layer of the dental epithelium (Fig. 1B), whereas it was also expressed in odontoblasts during tooth development (Fig. 1).

*In situ* hybridization analyses showed that fibronectin mRNA was expressed during the PS, S, and LM stages, but not during the EM stage (Fig. 1C). Fibronectin was detected in the basal lamina, odontoblasts, and ameloblasts during the PS stage, whereas its expression decreased in differentiated cells from the PS to EM stage. Fibronectin was also strongly expressed in ameloblasts during the LM stage. Real-time PCR was used to analyze the expression levels of amelogenin and fibronectin mRNA during each stage of differentiation in ameloblasts that were dissected from the incisors of newborn mice (Fig. 1D). The expression of fibronectin mRNA decreased from the PS to EM stage, whereas it was strongly detected during the LM stage. In contrast, amelogenin mRNA expression increased from the PS to S stage, and was not detected during the LM stage. Moreover, the level of amelogenin mRNA increased from the PS to S stage, and was not detectable during maturation. Expression levels of amelogenin and fibronectin mRNA were detected in the isolated dental epithelium and dental mesenchyme in P1 and P7 mouse molars (Fig. 1E). Fibronectin mRNA expression, but not that of amelogenin, was also detected in dental mesenchymal cells. Moreover, on P7, the expression of amelogenin mRNA was increased in dental epithelium cells, while that of fibronectin was decreased.

The results of immunostaining and *in situ* hybridization analyses were similar, and indicated that fibronectin was expressed in undifferentiated ameloblasts and odontoblasts, as well as in the LM stage of ameloblasts and papillary-layer cells.

### Expression of β Family Integrins in the Dental Epithelium and Mesenchyme

**Fig 2:**
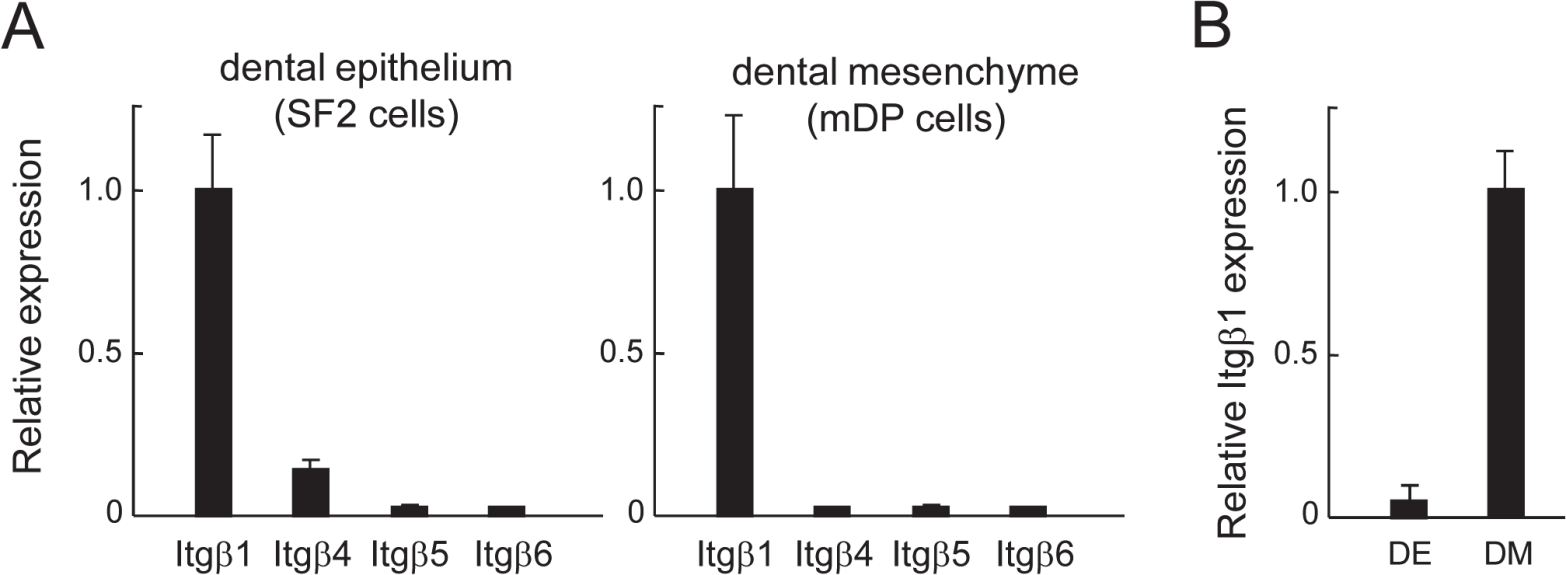
Expression of β family integrins in the dental epithelium and mesenchyme. A. Real-time PCR analysis of β family integrins in the dental epithelial cell line SF2 (left) and the dental mesenchymal cell line mDP (right). β1 integrin showed a higher expression level as compared to other integrins in both SF2 and mDP cells. B. Expression of β1 integrin in the dental epithelium (DE) and mesenchyme (DM) from P1 mouse molar tooth germs shown by real-time PCR. Integrin expression was higher in the DM as compared to the DE. PCR primers used are provided in the Materials and Methods section.

The major adhesion receptor for fibronectin is α5β1 integrin. To analyze the role of fibronectin in ameloblast differentiation, we examined the expression levels of β family integrins in SF2 cells derived from a dental epithelial cell line and in mDP cells derived from a dental mesenchymal cell line using real-time PCR. The most common form of β family integrins was β1 integrin, which was expressed in both SF2 and mDP cells (Fig. 2A). In SF2 cells, β4 integrin was weakly detected. However, epithelial β1 integrin expression was weaker in the dental epithelium as compared to that in the dental mesenchyme in real-time PCR analysis of P1 molar tooth germs (Fig. 2B).

### RGD Peptide and Antibody Against β1 Integrin Inhibited Binding of Dental Epithelial Cells to Fibronectin, Collagen I, and Laminin 511/521

**Fig 3:**
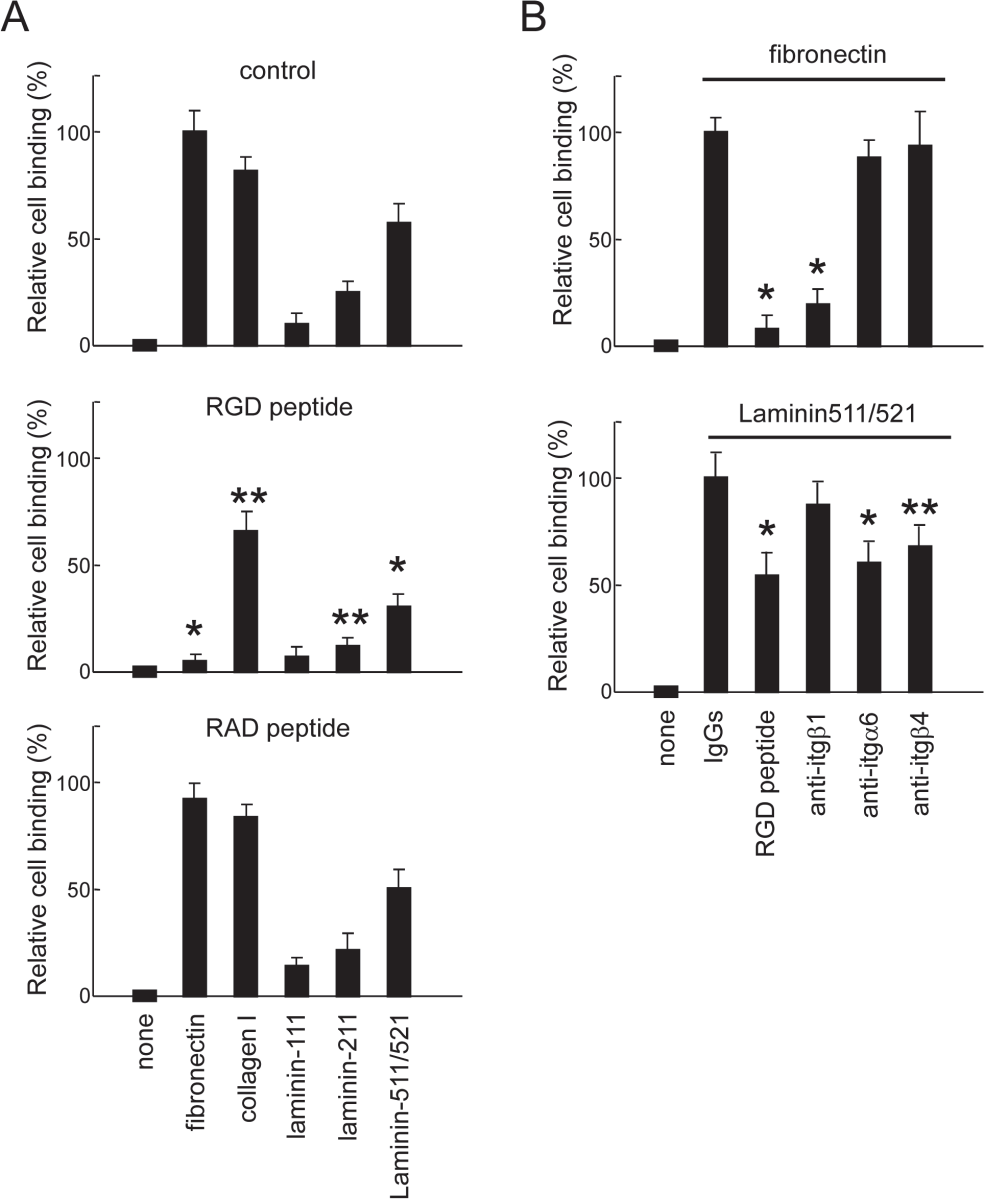
Dental epithelial cell adhesion assay. A. SF2 rat dental epithelial cells were cultured in plates coated with fibronectin, collagen I, mouse laminin 111 and 211, and human laminin 511/521 in the presence and absence of RGD or RAD peptides. Plates were stained with crystal violet. The relative number of adherent cells was evaluated using spectrophotometric analysis. B. SF2 cells were cultured in plates coated with fibronectin or laminin 511/521 in the presence and absence of IgGs, RGD peptide, and anti-β1, -α6, or -β4 antibodies. Cell adherence to fibronectin was inhibited by the RGD peptide and the anti-β1 integrin antibody, and adhesion of cells to plates coated with laminin 511/521 was inhibited in the presence of the RGD peptide, as well as anti-integrin α6 or anti-integrin β4 antibodies. *P < 0.01; **P < 0.05 (n = 6).

To investigate the roles of fibronectin and β1 integrin in the dental epithelium, SF2 cells were cultured in dishes coated with fibronectin, collagen I, or laminin, and then examined for their cell-adhesive properties (Fig. 3A). SF2 cells did not adhere well to plates coated with laminin 111 and 211, whereas they showed good adherence to plates coated with fibronectin, collagen I, or laminin 511/521. However, the RGD peptide inhibited the adherence of cells to plates coated with fibronectin, laminin511/521, or collagen I, indicating that dental epithelial cell adherence occurs via the RGD motifs of fibronectin, laminin 511/521, and collagen I. The adherence of SF2 cells to fibronectin was inhibited by an anti-β1 integrin blocking antibody. However, adhesion to laminin 511/521 was not inhibited by the same antibody, while inhibition was observed with the anti-integrin α6 and anti-β4 antibodies (Fig. 3B), suggesting that fibronectin binding occurs via the RGD domain of fibronectin and that β1 integrin contributes to epithelial cell adhesion.

### Partial Enamel Hypoplasia of the Cystic Epithelium during Terminal Differentiation of Ameloblasts in *Itgβ1*CKO Mice

**Fig 4:**
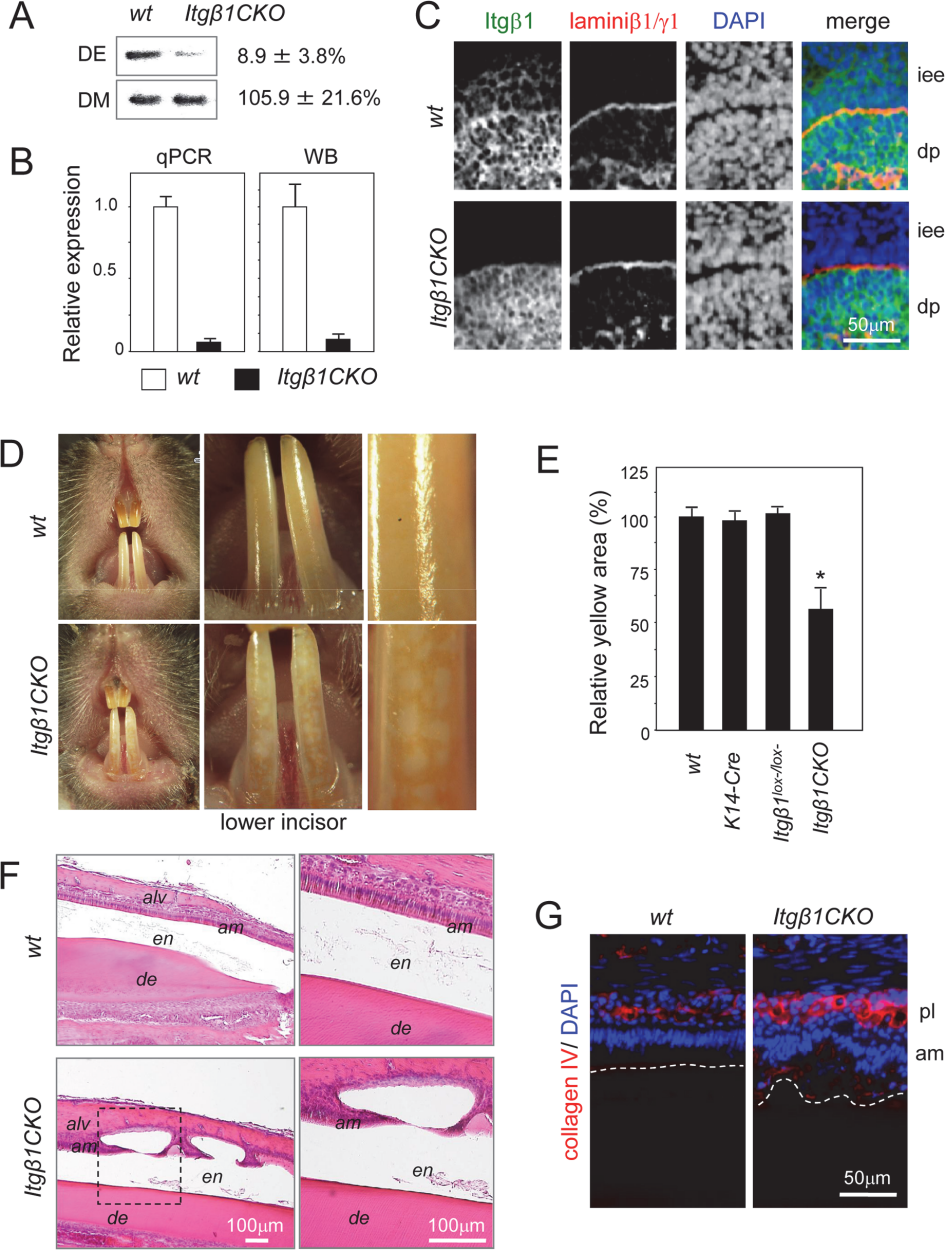
Phenotype of incisors from *Itgβ1*CKO mice. *Itgβ1* was deleted from cells that expressed cytokeratin 14. A. Southern blot analysis of genomic DNA from P1 molars using a probe for exon 3. Most of the region containing exon 3 was removed by Cre recombinase under the control of the *K4* promoter in the dental epithelium. B. Expression levels of β1 integrin mRNA and protein in P1 molar tooth germs. C. Immunohistolocalization of β1 integrin in E16.5 molar tooth germs. D. Frontal view of a 8-week-old wild type (WT) and homozygous *Itgβ1* conditional knockout (CKO) mouse. Many vitiligo patches are visible in the image of the lower incisor from an *Itgβ1*CKO mouse. E. The lower incisors were observed under higher magnification and the total size of the yellow areas was measured. Yellow areas were markedly decreased in the incisors from *Itgβ1*CKO mice (n = 5). F. Sagittal sections of lower incisors from a 6-week-old WT and *Itgβ1*CKO mouse were stained with hematoxylin and eosin (H-E). High magnifications of the areas enclosed by the dashed box in the left panel. Cysts were formed in the late maturation (LM) stage. G. Immunostaining using an anti-collagen IV antibody. Nuclei were stained using DAPI. The image shows an irregular and multilayer arrangement of ameloblasts in the incisor from an *Itgβ1*CKO mouse. The dotted line shows the border of enamel and ameloblasts. *P < 0.01. iee, inner enamel epithelium; dp, dental pulp; alv, alveolar bone; en, enamel; de, dentin; am, ameloblasts; pl, papillary layer.

To analyze the role of β1 integrin in tooth development, we used β1 integrin CKO mice under the control of *K-14-Cre*. Southern blot analysis showed reduction of the allele containing exon 3 in the *Itgβ1* ^lox-/lox-^/*K14-Cre* (*Itgβ1*CKO) mouse dental epithelium from P1 molar tooth germs, but not in the dental mesenchyme (Fig. 4A), and expression of β1 integrin mRNA and protein also decreased in the dental epithelium (Fig. 4B). Immunostaining for β1 integrin showed that it was specifically decreased in the dental epithelium, but not in dental mesenchyme in E16.5 molar tooth germs (Fig. 4C), indicating that this CKO mouse model is useful for analysis of the role of β1 integrin in the dental epithelium *in vivo*. *Itgβ1*CKO under the control of *K14-Cre* showed severe abnormalities of hair formation, similar to previous reports [14, 15]. The incisor of *Itgβ1*CKO mice were compared to that of WT mice (Fig. 4D), and many vitiligo patches were observed in the former. In addition, the number of the *Itgβ1CKO* mice with white spots in incisors increased (Fig. 4D and 4E), with 43 of 51 (84.3%) *Itgβ1*CKO specimens showing white spots. Because hypoplasia of the enamel generally presents as white teeth, we assumed that the phenotype of the *Itgβ1*CKO mice represented partial amelogenesis imperfecta. The sections at the tip of the incisors of a 6-week-old mouse were stained with H-E (Fig. 4F). Our results showed that ameloblast polarity was sustained, and smooth enamel parallel to the dentin was observed in the WT mouse specimens. On the other hand, ameloblasts in the *Itgβ1*CKO mouse specimens lost their polarity and were arranged in multilayers during maturation. Furthermore, we observed a cystic syrinx surrounded by nonpolar ameloblasts on the surface of the enamel (Fig. 4F). Prior to the expression of enamel matrices, collagen IV is expressed in the dental epithelium and forms the basement membrane. In the S and EM stages, collagen IV completely disappeared in the basal layer of ameloblasts (white dotted line in Fig. 4G) and was expressed in the papillary layer. Polarized ameloblasts were arranged along the strands of collagen IV, which was expressed in the papillary layer of the WT mouse specimens, whereas apolar and irregular arrangements of ameloblasts were observed in the *Itgβ1*CKO mouse specimens (Fig. 4G). These observations suggest that a cyst caused amelogenesis imperfecta and numerous white spots on the incisors of the *Itgβ1*CKO mice.

### β1 Integrin-null Ameloblasts Showed Delayed Differentiation During Maturation

**Fig 5:**
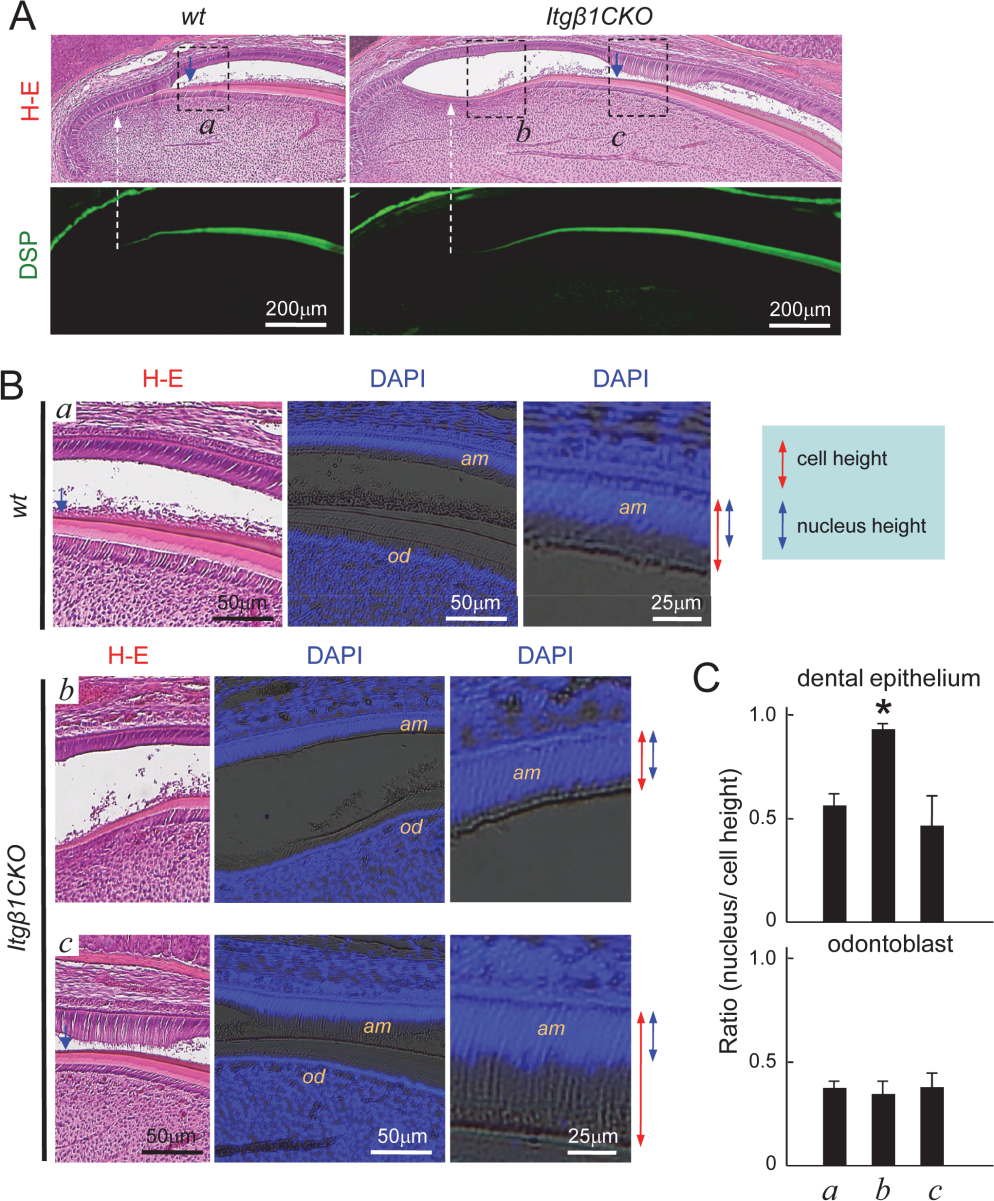
Sagittal sections of lower incisors from a 6-week-old WT and *Itgβ1*CKO mouse. A. The lower incisors of a wild type (WT) and a *Itgβ1* conditional knockout (CKO) mouse were stained with hematoxylin and eosin (H-E) and an anti-dentin sialoprotein (DSP) antibody. The white dotted arrow indicates the starting point of dentin formation from DSP-positive odontoblasts, and the blue arrow indicates the starting point of enamel formation from ameloblasts. Dentin (white dotted arrow) and enamel-formative regions (blue arrow) of the *Itgβ1*CKO-mouse incisors were separated by a longer distance as compared with those from the WT mice. B. High magnification of the area enclosed by the dotted box (a, amelogenesis origin of WT; b, dentinogenesis origin of *Itgβ1*CKO; c, amelogenesis origin of *Itgβ1*CKO) in Fig. 5A (DAPI staining). C. Ameloblast cell and nuclear heights are indicated by the red and blue arrows, respectively. The ratios of cells to nuclear heights are shown in the graph. The heights of the nuclei of undifferentiated ameloblasts did not change in the dentinogenesis region, and the ameloblasts became polarized and matured in the amelogenesis area. *P < 0.01. am, ameloblasts; od, odontoblasts.

To confirm our tentative diagnosis of amelogenesis imperfecta, the sagittal sections of a 6-week-old mouse incisors were analyzed using H-E staining and immunostaining of DSP. Amelogenesis (blue arrow in Fig. 5A) was initiated in close proximity to the initiation of dentin formation (white dotted arrow in Fig. 5A) in WT mice, because DSP is expressed by pre-odontoblasts before dentin formation, indicating that enamel and dentin are synchronously formed. However, the origin of integration was significantly delayed between enamel and dentin, because the enamel was formed after dentinogenesis in the *Itgβ1*CKO mice. High magnification of the dentin formation area revealed developed ameloblast differentiation, because WT cells were polarized (Fig. 5B). Although the polarity of the ameloblasts in the *Itgβ1*CKO mouse specimen did not occur at the start of dentin formation, it did develop at a point later than that observed in WT cells (Fig. 5B). Moreover, the ratio of the height of the cytoplasm to that of the nucleus was determined for each stage of cell polarization (Fig. 5C). Nuclear localization was not observed in dental epithelial cells of the *Itgβ1*CKO specimen at the start of dentin matrix secretion. Finally, polarization of the dental epithelium in the *Itgβ1*CKO specimen caught up at a later stage. These findings indicate that differentiation of ameloblasts was delayed in the absence of β1 integrin expression.

### Delay of Ameloblast Differentiation in *Itgβ1*CKO Mice

**Fig 6:**
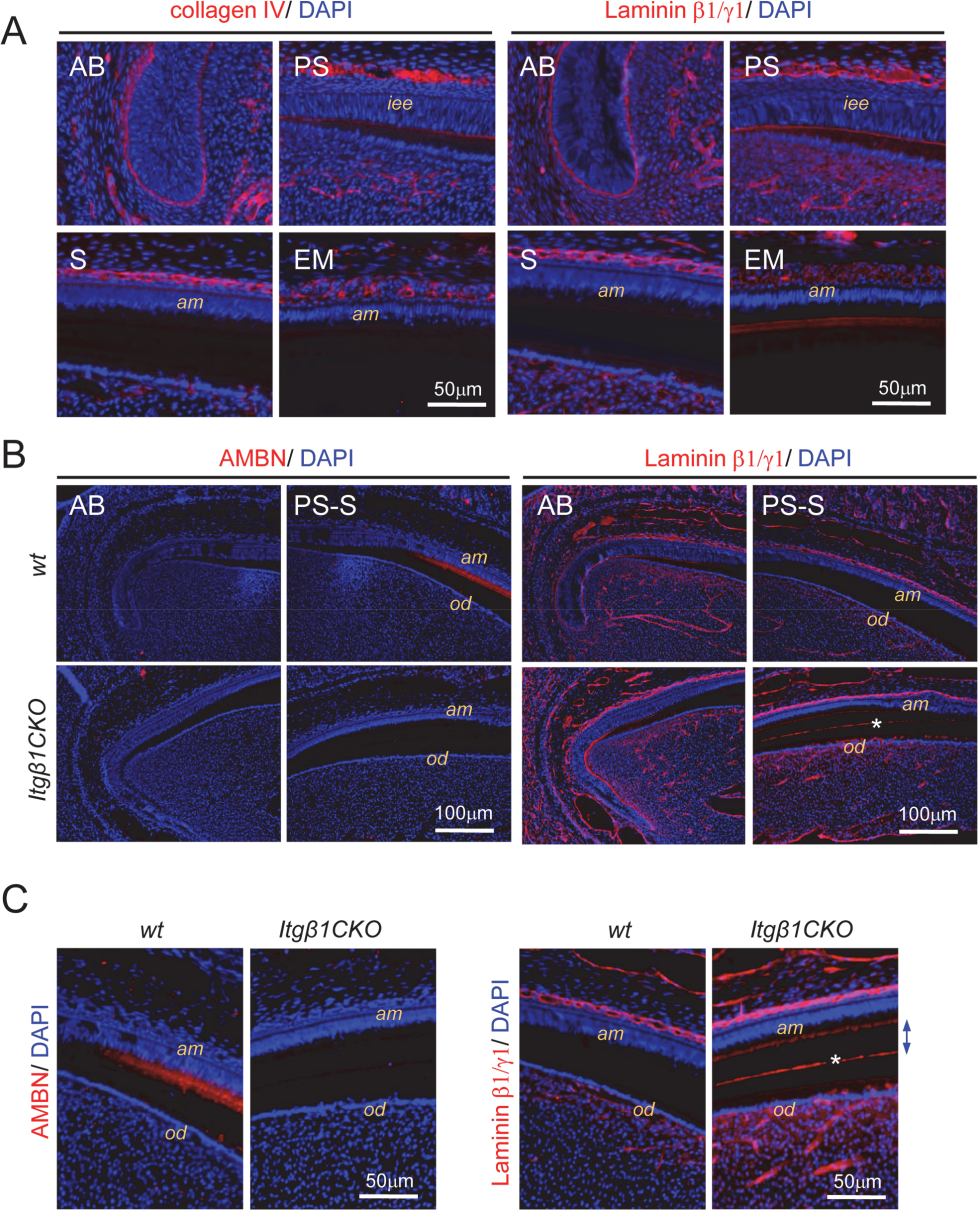
Expression of collagen IV, laminin β1γ1, and ameloblastin in incisors from WT and *Itgβ1*CKO mice. A. The lower incisor of a wild type (WT) mouse was analyzed using immunostaining with anti-collagen IV and anti-laminin β1γ1 antibodies. Laminin β1γ1 was expressed in the basal membrane, papillary layer, and immature ameloblasts. B, C. The lower incisors of WT and *Itgβ1* conditional knockout (CKO) mouse were immunostained by anti-ameloblastin and anti-laminin β1γ1 antibodies. Ameloblastin was detected in the incisor of a WT but not a *Itgβ1*CKO mouse. Laminin β1γ1 was detected in *Itgβ1*CKO (asterisk) but not WT incisors. iee, inner enamel epithelium; am, ameloblasts; od, odontoblasts.

Collagen IV was expressed in the boundary basal membrane of the epithelium and mesenchyme in the incisal apical area, and was also detected in the papillary layer during ameloblast maturation (Fig. 6A). Laminin β1γ1 was detected in the basal membrane, the papillary layer, and undifferentiated ameloblasts, but was undetectable during maturation. Next, we analyzed the expression levels of ameloblastin and laminin β1γ1 in incisors from WT and *Itgβ1*CKO mice (Fig. 6, B and C). Ameloblastin expression was detected in mature, but not immature WT ameloblasts and was not detected in the same tissues from the *Itgβ1*CKO mice. In contrast, laminin β1γ1 expression was detected in *Itgβ1*CKO ameloblasts, whereas expression was undetectable in the WT specimen, because the area of expression was extended (Fig. 6, asterisk in B and C). These findings indicate that ameloblast differentiation is delayed in the absence of β1 integrin.

### Delay of the Formation and Eruption of the Third Molar in *Itgβ1*CKO Mice

**Fig 7:**
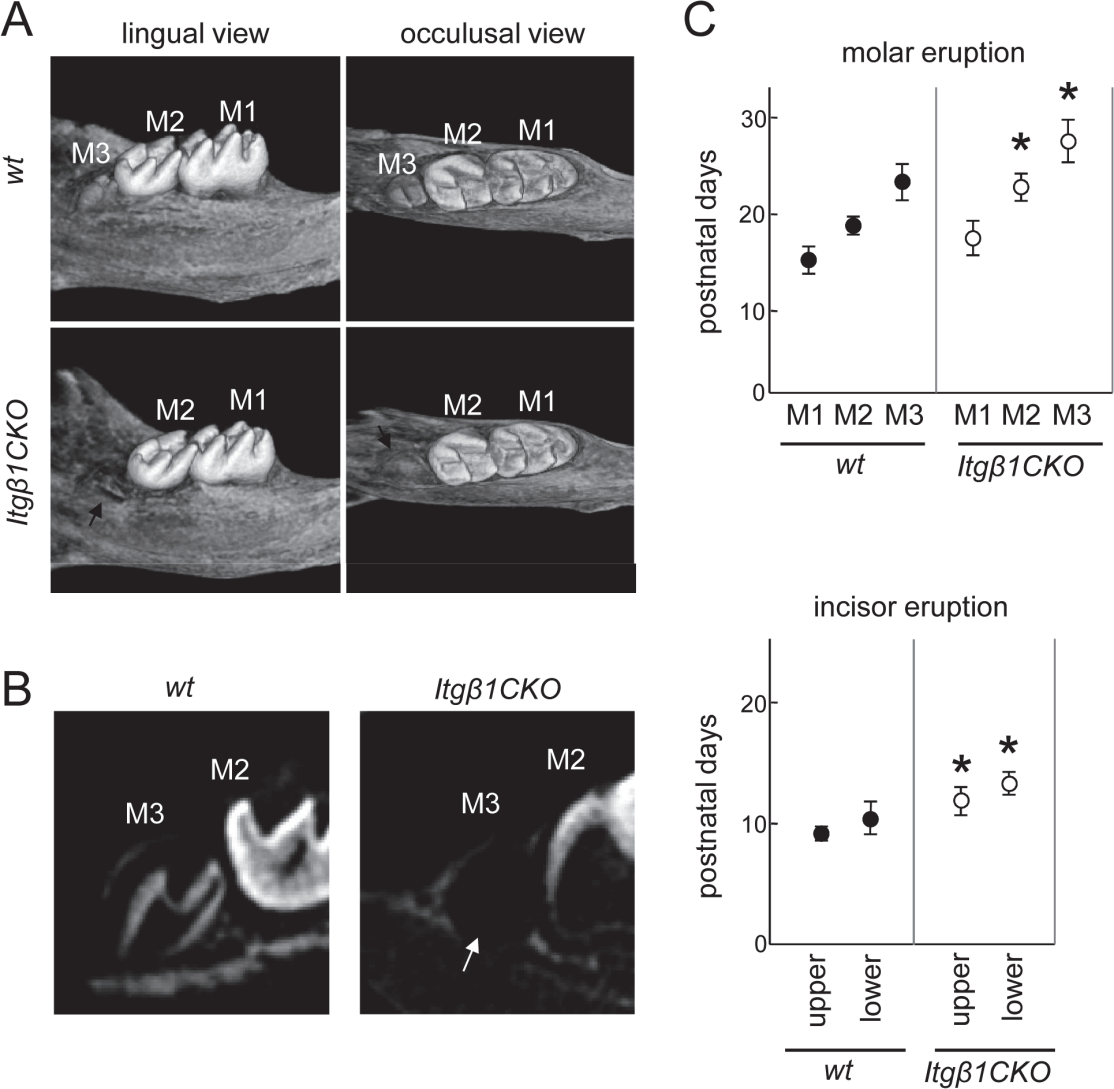
Eruption of lower third molars in P3 mice. The left mandible of a P3 mouse imaged using micro-computed tomography. A three-dimensional image was reconstructed from the lingual and occlusal sides. B. Image of the sagittal section of the third molar. Calcification and eruption of the third molar was not observed in the *Itgβ1* conditional knockout (CKO) mouse (white arrow). C. Lower molar and incisor eruption times. Eruption of the second and third molars and the incisor in the *Itgβ1*CKO mice was delayed. M1, first molar; M2, second molar; M3, third molar. *P < 0.01 (n = 10).

The mandible of a 3-week-old mouse is displayed as a three-dimensional image acquired using micro-CT in Fig. 7A. Eruption of the third molar was observed in the WT but not in the *Itgβ1*CKO mice (black arrow in Fig. 7A). A tomogram of the mandible was also imaged using micro-CT (Fig. 7B). Calcification of the third molar crown was observed in the WT but not in the *Itgβ1*CKO specimens, although the dental sac was present (white arrow in Fig. 7B). Incisor and molar eruption times in the WT and *Itgβ1CKO* mouse specimens are shown in Fig. 7C. The first molar erupted in both mice, although delayed eruption of the second and third molar and incisor was conspicuous in the *Itgβ1*CKO specimens, indicating that the delay in tooth eruption in the absence of β1 integrin reflected the delay in the differentiation of ameloblasts.

### Ameloblasts of *Itgβ1*CKO Mice Did Not Bind to Fibronectin and Expressed Decreased Levels of Ameloblastin

**Fig 8:**
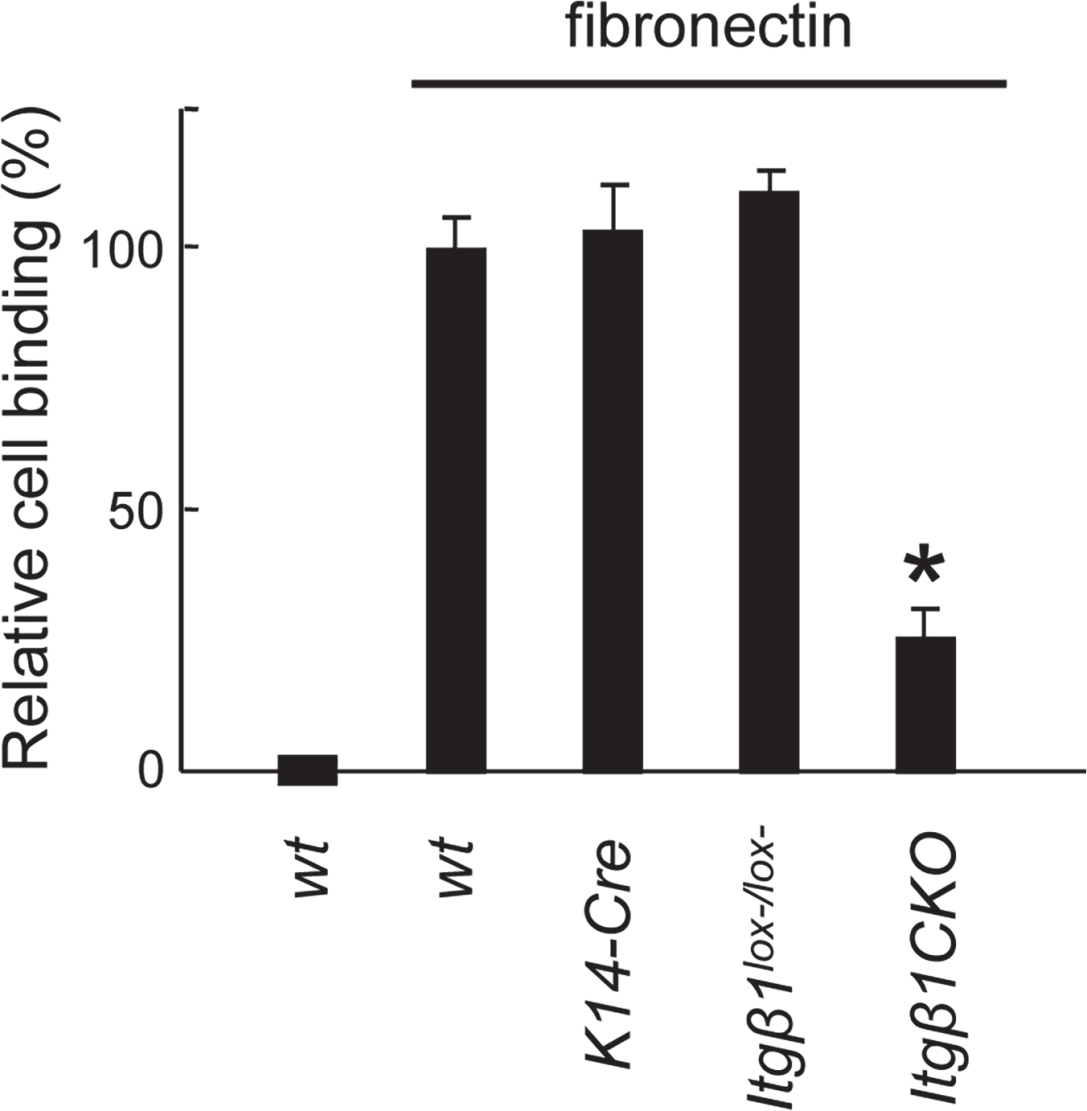
Analysis of ameloblast adhesion to fibronectin. Ameloblasts purified from wild type (WT), *K14-Cre*, *Itgβ1* ^*lox-/lox*-^, and *Itgβ1*CKO mice were cultured in fibronectin-coated dishes. The relative number of adherent cells stained with crystal violet was determined. Ameloblasts of *Itgβ1*CKO mice did not bind to fibronectin in contrast to those of WT, *K14-Cre*, and *Itgβ1* ^*lox-/lox-*^ mice. *P < 0.01 (n = 6).

The RGD motif of fibronectin binds to β1 integrin expressed by SF2 cells (Fig. 3). Therefore, dental epithelial cells from the *Itgβ1*CKO mouse were used instead of the SF2 cell line for binding assays (Fig. 8). Epithelial cells from WT, *K14-Cre*, and *Itgβ1*CKO specimens adhered to plates coated with fibronectin, while adhesion of epithelial cells from the *Itgβ1*CKO specimens was significantly lower.

**Fig 9:**
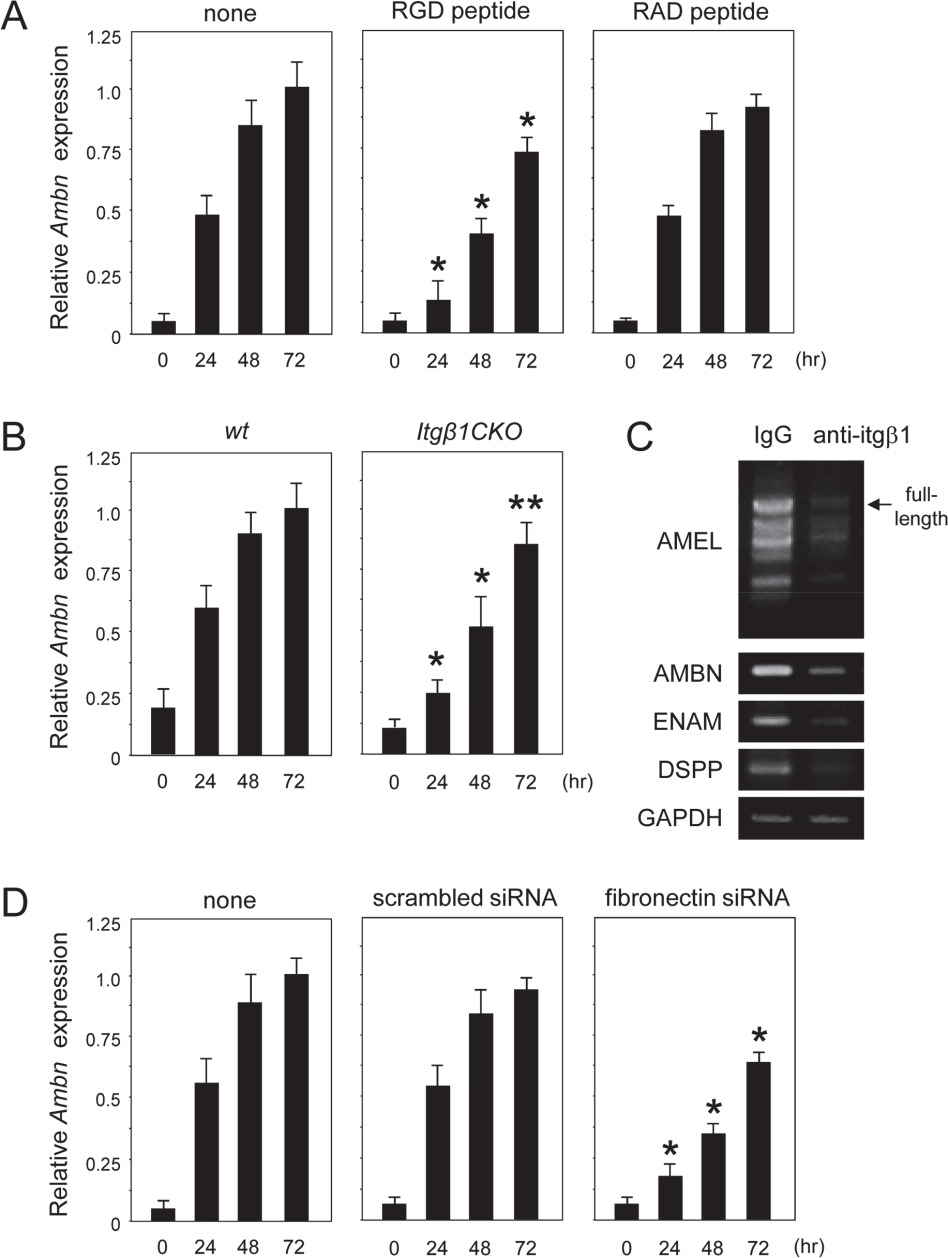
Real-time PCR analysis of expression of mRNAs encoding ameloblast markers in primary cultures of ameloblasts. A. Wild type (WT) ameloblasts were cultured with RGD or RAD peptide for 0 to 72 hours. The expression of ameloblastin was delayed by RGD but not by RAD. B. WT and *Itgβ1*CKO ameloblasts were cultured for 0 to 72 hours. The expression of ameloblastin was delayed in *Itgβ1*CKO ameloblasts as compared with WT. C. Analysis of the expression of mRNAs encoding ameloblast markers in WT ameloblasts cultured in the presence of an anti-β1 integrin antibody. The expression of all ameloblast markers was decreased. D. WT ameloblasts were transfected with fibronectin siRNA for 0 to 72 hours. The inhibition of fibronectin expression delayed the expression of ameloblastin. *P < 0.01; **P < 0.05 (n = 5).

Because the differentiation of ameloblasts was delayed in *Itgβ1*CKO mice, we determined the influence of cell adhesion on ameloblast differentiation. Previously, neurotrophic factor NT-4 was shown to induce ameloblastin expression [24]. Primary culture of dental epithelial cells was performed without a fibronectin coating. In this condition, the expression of ameloblastin after stimulation with NT-4 increased with time in primary epithelial cells but decreased in the presence of the RGD peptide (Fig. 9A). Furthermore, a delay in increased expression of ameloblastin was detected in epithelial cells of the *Itgβ1*CKO mouse specimens (Fig. 9B) and was inhibited when cells were transfected with a fibronectin siRNA (Fig. 9D), indicating that endogenous fibronectin is important for ameloblast differentiation. Expression levels of the ameloblast differentiation markers ameloblastin, enamelin, and dentin sialophosphoprotein, as well as each splice variant of amelogenin were decreased in ameloblasts when treated with the anti-Itgβ1 antibody (Fig. 9C), but not with the control IgG. These results indicate that the delayed differentiation was induced by inhibiting the interactions of ameloblasts with β1 integrin and fibronectin.

### Fibronectin Enhanced Neurotrophic Factor NT-4 and Induced ERK1/2 Phosphorylation

**Fig 10:**
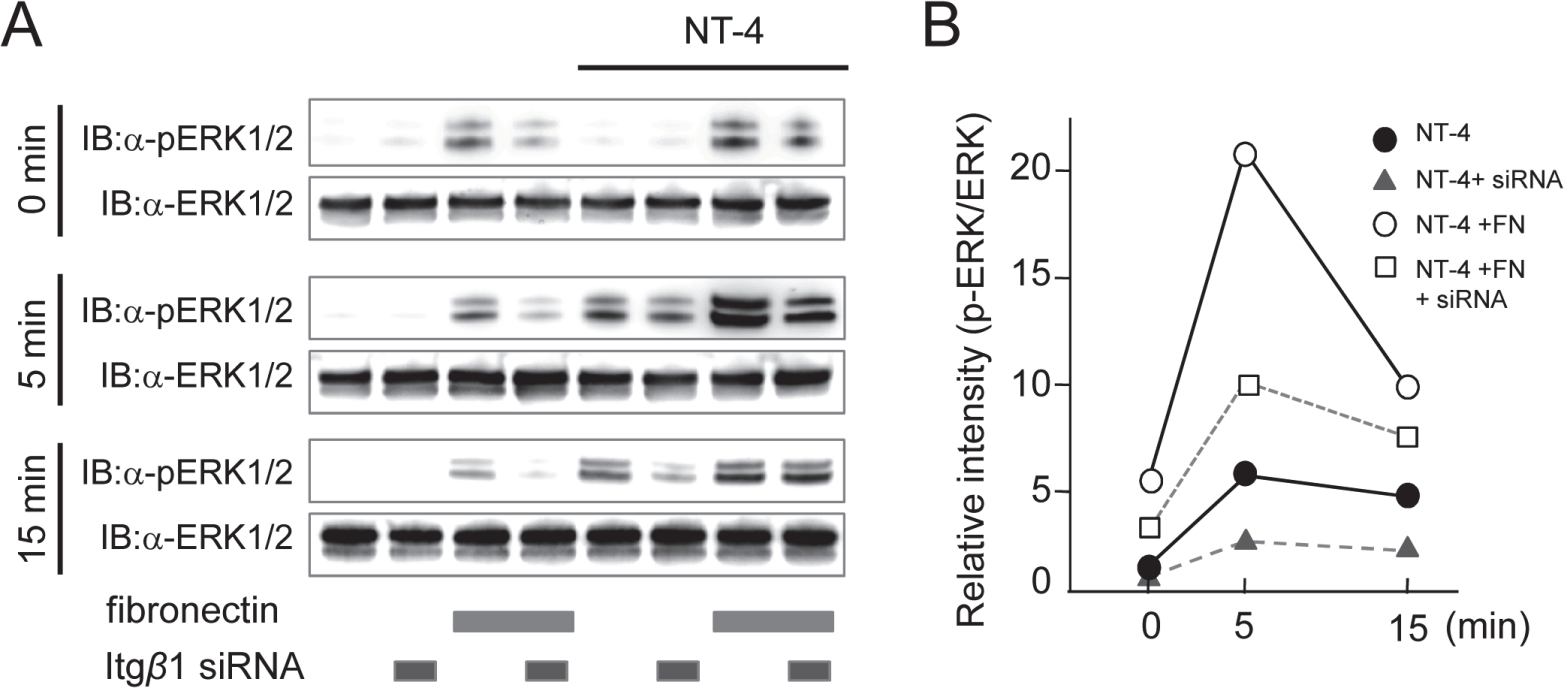
Phosphorylation of ERK1/2 after stimulation of neurotrophic factor NT-4 in the presence of fibronectin. A. Western blot analysis was performed using anti-ERK1/2 and phospho-ERK1/2 antibodies. Cell lysates were prepared after stimulation of NT-4 at 0, 5, and 15 minutes with or without fibronectin or an siRNA for β1 integrin. B. Quantitative data of ERK1/2 phosphorylation.

Ameloblastin expression was previously shown to be induced by NT-4 via ERK1/2 phosphorylation and was inhibited by the MEK inhibitor PD98059 [22, 24]. To determine fibronectin and β1 integrin integration in ameloblastin expression induced by NT-4, we examined the phosphorylation of ERK1/2 after stimulation of NT-4 with or without fibronectin. In cultures performed in fibronectin-coated dishes, ERK1/2 phosphorylation was detected in SF2 cells without NT-4 and was also observed at 5 minutes after NT-4 stimulation, and then enhanced in the presence of fibronectin (Fig. 10). To examine the role of integrin in the enhanced phosphorylation of ERK1/2 induced by fibronectin, we inhibited β1 integrin expression using an siRNA for β1 integrin. siRNA transfection decreased β1 integrin expression by about 72% (data not shown). In this condition, ERK1/2 phosphorylation was induced by NT-4 and was enhanced in the presence of fibronectin (Fig. 10). These results indicated that NT-4 induces ERK1/2 phosphorylation, while ameloblastin expression is regulated by the interaction between fibronectin and β1 integrin.

## Discussion

Fibronectin is a multifunctional glycoprotein that is synthesized in the mesenchyme by fibroblasts, hepatocytes, and endothelial cells [25, 26]. During tooth development, we found that fibronectin is expressed in the mesenchymal basal lamina by pre-ameloblasts during the PS stage, and by ameloblasts in the P and LM stages (Fig. 1). However, fibronectin expression was not detected during the EM stage, indicating a biphasic expression pattern in the various tooth development stages.

Fibronectin is essential for the initiation of epithelial branching in organs such as the salivary glands, lungs, and kidneys [27]. Furthermore, it creates a microenvironment in which pigment epithelial cells proliferate and induce wound healing [28]. The function of fibronectin in epithelial cells is gradually coming to light. Fibronectin binds to members of the αV subfamily, including α4β1, α4β7, α5β1, α8β1, and α9β1 integrins, via its RGD domain [29]. Our present results confirmed that fibronectin binds to β1 but not to β4 or α6 integrin, confirming the results of these previous studies.

The integrin isoforms α3, α5, α6, αV, β1, β4, β5, and β6 integrins are expressed by the dental epithelium [17, 29–31], indicating that β1 integrin, which showed a higher expression level as compared to β4, β5, and β6, is the most important integrin molecule that binds to fibronectin in the dental epithelium. We attribute this retardation of tooth growth to a delay in ameloblastin expression in ameloblasts (Fig. 9). The phenotype of ameloblastin-null (*Ambn* ^-/-^) mice is characterized by a delay in cell differentiation but not tooth eruption [20]. Ameloblastin is expressed from the S stage to the early maturation stages. In contrast, β1 integrin is expressed in the epithelium and mesenchyme early during tooth development [32]. Therefore, β1 integrin might have effects on the expression of ameloblastin.

Delays in establishing cell polarity and eruption also occurred in *Itgβ1*CKO mice. Previous research showed that delayed molar eruption does not occur in *Itgβ6* ^-/-^ mice, although cell adhesion is decreased and expression of enamel matrix protein is increased [30]. In contrast, we found that the expression of enamel matrix protein was decreased in *Itgβ1*CKO mice (Fig. 9). Therefore, the mechanisms of amelogenesis imperfecta in *Itgβ6*KO and *Itgβ1*CKO mice may differ. Molar eruption is also delayed in *Osx-Cre;Tgfbr2(fl/fl)* mice when the gene encoding the TGF-β receptor II is deleted, which is attributed to defects in root formation, including failure of the root to elongate [33]. Moreover, delayed molar eruption occurs in mice with osteopetrosis induced by mutations in the genes encoding macrophage colony-stimulating factor, msh homeobox 2, and receptor activator of nuclear factor kappa-B, as well as in mice with cleidocranial dysplasia [34–37]. Thus, delayed eruption of molars is caused by abnormal bone and tooth root integration. Because the conditional deletion of *Itgβ1* in *Itgβ1*CKO mice is limited to the epithelium under control of the *K14* promoter, the phenotype was strongly expressed in the epithelium. The delay in tooth eruption can be attributed to the delay in differentiation of the dental epithelium (e.g., delays in cell polarity and ameloblastin expression) caused by loss of cell adhesion mediated by the fibronectin-β1 integrin complex.

The present results establish a link between fibronectin, collagen I, and laminin 511/521 and adhesion of ameloblasts (Fig. 3A). Type I collagen regulates the genes encoding dentin matrix protein 1, osteocalcin, alkaline phosphatase, and bone sialoprotein in dental pulp cells [38, 39]. Osteogenesis imperfecta, caused by mutation of the gene encoding collagen I, induces dentine dysplasia [40]. Therefore, collagen I is required for dentin formation. Laminin 511 and 521 contain laminin α5, and play an important role in determining the size and shape of tooth germs through proliferation, and the polarity of basal epithelial cells [1]. In our experiments, *Itgβ1*CKO mice showed partial enamel hypoplasia, although the phenotype was milder than originally speculated. In this mouse line, other molecules, including those related to matrices, laminin, and collagen IV, may support ameloblast differentiation.

Fibronectin induces the differentiation of dental pulp cells to mineralized tissue-forming cells for the repair of dentine and pulp via contact with cells [41, 42]. However, the effects of fibronectin on dental epithelial cells, including ameloblasts, have not been clearly reported. We found that the interaction of fibronectin and β1 integrin via the RGD motif is important for ameloblast adhesion (Figs. 3, 8). Furthermore, this interaction regulates NT-4-induced ERK1/2 phosphorylation and ameloblastin expression (Figs. 9, 10). In mammals, at least 24 distinct integrin receptors are formed from 18 α and 8 β subunits, including the β1 integrin and β7 subfamily members that bind to fibronectin via the RGD motif [43–46]. In contrast, β3 and β6 integrins are expressed during maturation [47]. In β3-deficient mice, although pigmentation is lacking because of iron transport inhibition, the enamel was shown to be normally formed and the level of ameloblastin expression was not altered [48].

Integrin β6-null mice have enamel malformations that mimic human amelogenesis imperfecta [30]. Mutations in the human genes encoding integrin α6 and integrin β4 (integrin α6β4) cause junctional epidermolysis bullosa with pyloric atresia, which includes enamel defects [49]. In addition, conditional knockout of β1 integrin in the mouse dental epithelium causes altered tooth morphogenesis [32]. However, the mechanism of abnormal morphogenesis is not clear. Our results indicate that the mechanism of amelogenesis imperfecta in *Itgβ1*CKO mice is cyst formation because of obliteration of induced mature ameloblast cell polarity during the terminal stage of differentiation caused by decreased cell adhesion. We speculate that the absence of β1 integrin causes a cyst and amelogenesis imperfecta by inducing remarkable inhibition of cell adhesion, because β1 integrin forms complexes with fibronectin during the maturation stage.

Laminin and fibronectin mediate the migration and attachment of the junctional epithelium [50], while fibronectin is also involved in adhesion to the periodontal ligament [51]. Although ameloblastin participates in the adherence of ameloblasts, the expression of ameloblastin is decreased with quenching of the basement membrane in a maturity-related manner. Therefore, fibronectin may be important for cell adhesion in the terminal maturation stage of ameloblasts
